# Influences of Recycled Polyethylene Terephthalate Microplastic on the Hygrothermal and Mechanical Performance of Plasterboard with Polymethylhydrosiloxane Content

**DOI:** 10.3390/ma17071652

**Published:** 2024-04-03

**Authors:** Victoria Romano-Matos, Alain Tundidor-Camba, Sergio Vera, Ivan Navarrete, Alvaro Videla

**Affiliations:** 1 Department of Mining Engineering, Pontificia Universidad Catolica de Chile, Vicuña Mackenna 4860, Santiago 8320000, Chile; veromano@uc.cl; 2 Physical Chemistry Department, Faculty of Chemistry and Pharmacy, Pontificia Universidad Catolica de Chile, Vicuña Mackenna 4860, Santiago 8320000, Chile; atundido@uc.cl; 3 Research Laboratory for Organic Polymers (RLOP), Department of Organic Chemistry, Pontificia Universidad Catolica de Chile, Santiago 8320000, Chile; 4 UC Energy Center, Pontificia Universidad Catolica de Chile, Vicuña Mackenna 4860, Santiago 8320000, Chile; svera@uc.cl; 5 Department of Construction Engineering and Management, Pontificia Universidad Catolica de Chile, Vicuña Mackenna 4860, Santiago 8320000, Chile; ivan.navarrete@uc.cl; 6 Center for Sustainable Urban Development CEDEUS, Pontificia Universidad Catolica de Chile, Vicuña Mackenna 4860, Santiago 8320000, Chile; 7 Concrete Innovation Hub UC (CIHUC), Pontificia Universidad Catolica de Chile, Av. Vicuña Mackenna 4860, Santiago 7820436, Chile

**Keywords:** water absorption, flexural strength, thermal conductivity, morphology, porosity, polyethylene terephthalate, polymethylhydrosiloxane, hygrothermal and mechanical performance

## Abstract

New composites produced with recycled waste are needed to manufacture more sustainable construction materials. This paper aimed to analyze the hygrothermal and mechanical performance of plasterboard with a polymethylhydrosiloxane (PMHS) content, incorporating recycled PET microplastic waste and varying factors such as PMHS dose, homogenization time, and drying temperature after setting. A cube-centered experimental design matrix was performed. The crystal morphology, porosity, fluidity, water absorption, flexural strength, and thermal conductivity of plasterboards were measured. The results showed that incorporating recycled PET microplastics does not produce a significant difference in the absorption and flexural strength of plasterboards. However, the addition of recycled PET reduced the thermal conductivity of plasterboards by around 10%.

## 1. Introduction

Plasterboard consists of a hardened gypsum core between sheets of paper on each side. Natural gypsum, or calcium sulfate dihydrate (CaSO_4_·2[H_2_O]), is widely used as a lightweight construction material in different types of products due to its low cost and ease of installation [1]. The microstructure of plasterboards has a high porosity and high internal surface area due to interlocking crystals. The characteristics of the material’s microstructure are responsible for its physical properties [2]; for example, properties like pore content, water solubility, and crystal size can have an effect on water affinity [3].

The manufacturing process of CaSO_4_·2[H_2_O] includes the following stages [4]. First, it must be dehydrated by heating, causing a phase change from CaSO_4_·2[H_2_O] to calcium sulfate hemihydrate (CaSO_4_·0.5[H_2_O]). Next, to prepare a pourable paste, CaSO_4_·0.5[H_2_O] is mixed with water and other admixtures, which is then poured onto a pre-sized sheet of paper or cardboard that is posteriorly molded. Finally, the molded board is set to harden and dry.

CaSO_4_·2[H_2_O] formation based on CaSO_4_·0.5[H_2_O] is a crucial manufacturing step. When water is added, CaSO_4_·0.5[H_2_O] hardens and remains in its original dihydrate state. Singh and Middendorf [5] and Chen et al. [6] found that CaSO_4_·0.5[H_2_O] hydration, leading to the formation of CaSO_4_·2[H_2_O], is due to the initial dissolution of CaSO_4_·0.5[H_2_O] particles in water, which leads to the precipitation of the less soluble CaSO_4_·2[H_2_O]. The particle size of CaSO_4_·0.5[H_2_O] controls the generation of mesopores; smaller particles promote a faster dissolution before the setting process [7]. Crystal size depends on the duration of the set stirring time during the formation of CaSO_4_·2[H_2_O] [8].

Li et al. [9] and Roveri et al. [10] found that the use of chemical additives could enhance the control of the final structure and properties resulting from CaSO_4_·2[H_2_O] formation based on CaSO_4_·0.5[H_2_O]. These studies showed that using a hydrophobic organic emulsion encased the CaSO_4_·0.5[H_2_O] particles during hydration, causing a change in the physical properties of the hydrated product; the result was a smoother surface of the product that prevented further hydration, and the crystal morphology of the intertwined CaSO_4_·2[H_2_O] changed from the classic needle structure to thicker and shorter column forms.

Interactions between CaSO_4_·0.5[H_2_O] and chemical additives depend on the nucleation process, which usually occurs through the blending of primary species by collision. However, chemical additives usually reduce the collision probability, resulting in more time for crystal nucleation and for the transformation into crystalline CaSO_4_·2[H_2_O] [5,11,12,13,14,15,16,17,18,19,20,21]. Additionally, Pan and Li [22] proposed the use of an admixture containing fluorinated silicone to improve the humidity resistance. They found that the microscopic pores were covered by a film provided by the waterproof agent, turning the internal macropore surface from hydrophilic to hydrophobic, resulting in increased water repulsion and strength due to the prevention of water penetration in the matrix.

Wu et al. [23] found that sodium methyl silanol increased water absorption by reducing the pore size (on average, from 500 μm to 100 μm) and increasing pore interconnectivity. Other researchers showed that some admixtures that act as hydration accelerators promote the formation of a denser and more compact crystal microstructure, boosting the impermeability of the CaSO_4_·2[H_2_O] matrix [23,24]. The type and dose of the additive, which influences the formation of hydration products and the final CaSO_4_·2[H_2_O] microstructure, ultimately influences the material’s porosity and could effectively reduce it. Furthermore, additives can be used to form fine structures, which are associated with higher mechanical strength and humidity resistance [24,25,26].

The most widely used chemical additive in plasterboard to provide moisture resistance is polymethylhydrosiloxane (PMHS) [27], giving the matrix of the plasterboard its hydrophobic property. PMHS is composed of a repetitive structure of silicon, oxygen, and hydrogen atoms and methyl groups linked by covalent bonds. The methyl groups are responsible for the high hydrophobicity of the material and reach a contact angle of 100 ± 2.0° on the plasterboard [27,28,29].

Several recent studies have been carried out to improve the properties of plasterboards by reusing waste material. For instance, previous efforts to add plastic waste to the CaSO_4_·2[H_2_O] matrix include the research conducted by Pedreño-Rojas et al. [30], who mixed recycled polycarbonate from waste compact discs (CDs) and digital versatile discs (DVDs) with CaSO_4_·0.5[H_2_O] and recycled CaSO_4_·2[H_2_O]. The mechanical strength increased due to the recycled CaSO_4_·2[H_2_O], which contained fiberglass remains, but the density and thermal conductivity decreased. Del Rio Merino et al. [31] incorporated ceramic waste and extruded polystyrene with the objective of reducing the amount of raw material and improving the properties of traditional CaSO_4_·2[H_2_O]. The results showed that the water absorption decreased, while the surface hardness increased compared to the reference CaSO_4_·2[H_2_O]. Santamaria-Vicario et al. [32] and Buggakupa et al. [33] carried out experiments using polyurethane foam residues and used CaSO_4_·2[H_2_O] molds and glass remains to produce water-resistant CaSO_4_·2[H_2_O]-based products. In addition, previous studies [34,35] on the use of plastic residues in CaSO_4_·2[H_2_O] matrices showed that the addition of plastic residues improved the surface hardness and absorption and significantly reduced CaSO_4_·2[H_2_O] and water consumption without affecting the hygrothermal properties, while keeping their mechanical characteristics above the minimums required under current regulations.

Furthermore, beyond the previous research mentioned regarding the reuse of waste material as additives, the research conducted by Zhu et al. [36] studied the effect of incorporating polyvinyl alcohol and polypropylene fibers into CaSO_4_·2[H_2_O]-based compounds to influence properties such as workability, hydration kinetics, flexural strength, and hardness. The results obtained demonstrated that the inclusion of fibers significantly increased the flexural strength and hardness, but decreased the workability and hydration rate of the samples. Furthermore, using scanning electron microscopy (SEM), it was observed that the interfacial transition zone (ITZ) between the fiber and the CaSO_4_·2[H_2_O] was remarkably compact, and the space was much smaller, which is relevant when analyzing the effect of the plastic–CaSO_4_·2[H_2_O] matrix union.

Some previous work has been carried out regarding the use of polyethylene terephthalate (PET). Ali et al. [37] studied the effect of incorporating lightweight PET waste into CaSO_4_·2[H_2_O] matrices under standard laboratory conditions and obtained improved physical, mechanical, and insulating performances. The research found that the best behavior was achieved in mixtures with an addition of 7% PET weight, while the flexural strength decreased by over 10%. In addition, Erdem and Arioglu [38] produced a composite material by adding recycled PET fibers and an additive that improved adhesion in the CaSO_4_·2[H_2_O] matrix and properties. The test results showed that adding the fibers slightly decreased the flexural strength, but the admixture improved adhesion, producing less reduction in the flexural and compressive strength. Additionally, in another study, mixtures were used to analyze the influence of different amounts of solid residues such as recycled PET on the mechanical properties of CaSO_4_·2[H_2_O] at room temperature. Adding this residue to CaSO_4_·2[H_2_O] improved the compression strength compared to the reference mix [39].

Some research has also been undertaken to evaluate the use of different fillers and fine materials on hydrated calcium sulfate preparations. Doleželová et al. [40] studied the structure and behavior of CaSO_4_·2[H_2_O] compounds prepared with different fillers and fine materials such as silica sand, perlite, expanded clay aggregate, and residual polyurethane foam and the mechanical strength, thermal conductivity, and moisture were measured. The intrinsic properties of the aggregate type and their surface quality were found to affect the CaSO_4_·2[H_2_O] crystal size and shape significantly. Flexural strength increased with a higher surface roughness of the particles. The more porous the particle surface, the smaller the CaSO_4_·2[H_2_O] crystals in the ITZ and the more densely packed they were.

We can see that these conclusions are similar to the previously mentioned investigations regarding the properties of plasterboard with added plastics. In general, the water absorption capacity, the thermal conductivity, and the mechanical strength decreases [34,41] as the plastic weight percentage in the mixture increases. However, none of these investigations included the evaluation of the interaction between the effect of plastic waste and the preparation variables such as stirring time, PMHS dosage, and drying temperature after the setting of CaSO_4_·2[H_2_O]. Such an understanding could allow us to improve our understanding for the industrial preparation of plasterboards.

In addition, in our previous study [42], we evaluated the effect of PMHS on the morphology and porosity of CaSO_4_·2[H_2_O] plasterboard. The results showed that the PMHS admixture caused changes in the morphology and porosity of the CaSO_4_·2[H_2_O] structure obtained, which decreased the moisture absorption and thermal conductivity without affecting the flexural strength.

In this investigation, we chose to evaluate the effect of a fine microplastic PET as a material filler in plasterboard. The reutilization of fine microplastics as a filler could help with the recirculation of a material not useful for recycling. The fine microplastic waste resulting from the plastic recycling process is usually sent to a final disposal landfill where it will stay for decades. This material is not selected to be recycled because it reduces the performance of the recycling process and generates operational problems in the whole recycling plant. These microplastics are flakes obtained after grinding and screening while the PET is prepared for recycling, and they have a particle size smaller than 5 mm. The addition of these recycled microplastics may cause positive changes in the morphology and porosity of plasterboard, affecting the water absorption, flexural strength, and thermal conductivity. To evaluate these effects, several tests were carried out under controlled conditions to which the boards were subjected during the manufacturing process. The plasterboard performance is related to the effects of the PMHS dosage, stirring time of the mixture, and the drying temperature after setting. This research contributes to improving our understanding of the effect of the addition of fine particles of PET to plasterboard and to the creation of a circular economy where PET can be used in plasterboard production.

## 2. Materials and Methods

### 2.1. Materials Characterization

**Figure 1:**
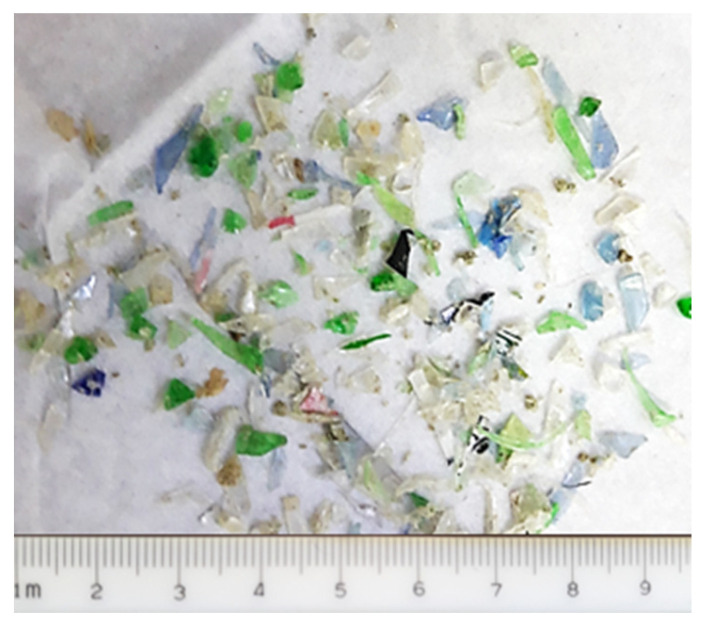
Sample of the PET microplastic.

In this study, the following materials were used: CaSO4·0.5[H_2_O], PMHS, and recycled PET microplastics. The calcium sulfate beta hemihydrate of natural CaSO4·0.5[H_2_O] complied with the ASTM C28/C28M, 2010 Specification for Gypsum Plasters [43]. The PMHS used was a sample of the Elkem Silicones France Bluesil WR 68 product. The recycled PET microplastic in the form of flakes smaller than 5 mm was obtained via sieving ground PET particles in the industrial mechanical recycling process. The plastic particles are shown in Figure 1. It is important to highlight that these particles are currently discarded in landfills.

Elemental characterization of CaSO_4_·0.5[H_2_O] was obtained with X-ray fluorescence (XRF), which is shown in Table 1. The device used was a WDX S4 TSTAR Bruker wavelength dispersive sequential spectrometer. To quantify the oxide forms of the compounds, the samples were calcined at 1050 °C for 150 min. After calcination, the molten sample was placed in the sample holder of the analytical equipment. The fusion was carried out with M4 Claisse equipment, producing a uniform, vitreous disc. In addition, the mineralogical composition of the calcium sulfate hemihydrate was measured via X-ray diffraction (XRD), which is shown in Table 2.

The particle size distribution (PSD) of CaSO_4_·0.5[H_2_O] was measured with laser ray scattering in a liquid suspension using a laser diffraction analyzer (LDA), specifically the Mastersizer 2000, Malvern Instruments Ltd. Enigma Business Park, Grovewood Road, Malvern, Worcestershire WR14 1XZ United Kingdom.

**Figure 2:**
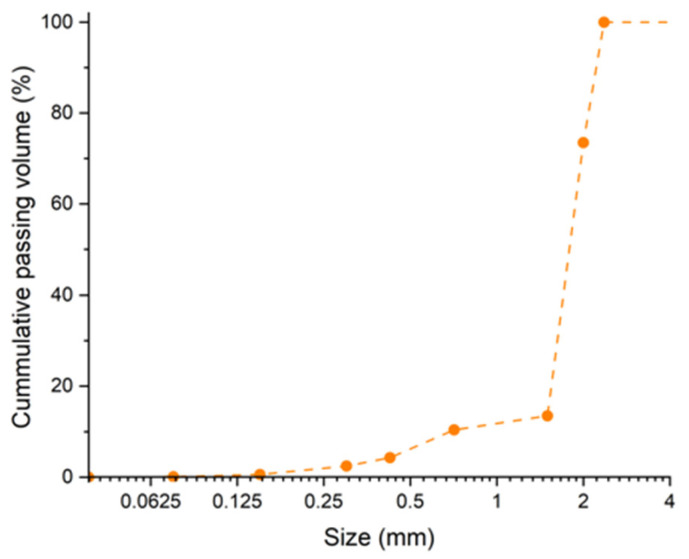
Particle size distribution of the recycled PET microplastic.

Figure 2 shows the particle size distribution of microplastics resulting from PET product recycling. The original sample size was 60% smaller than 2.36 mm.

**Figure 3:**
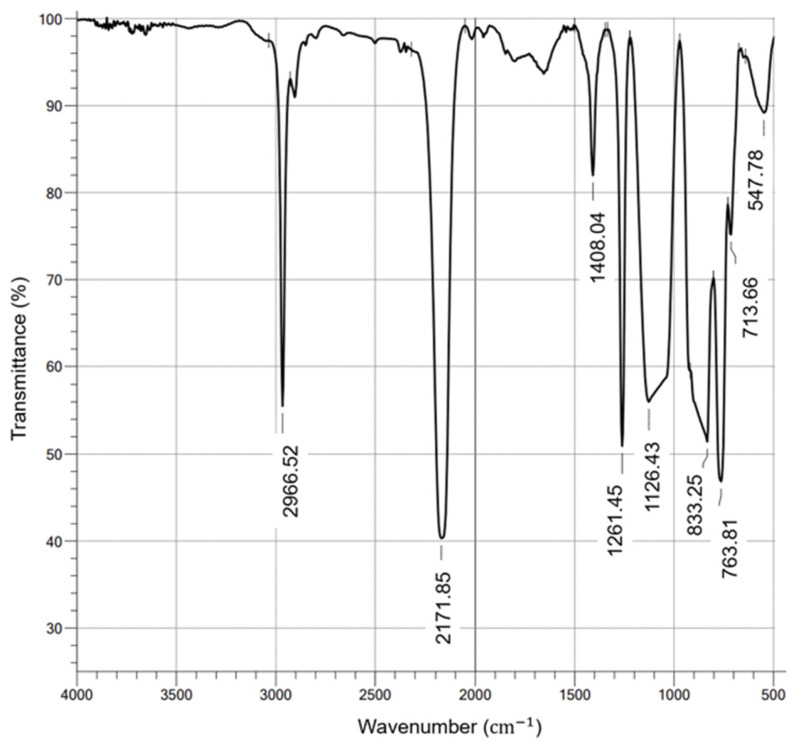
Infrared spectra of PMHS.

On the other hand, the PHMS was characterized based on the functional groups, which were determined with a Shimadzu brand infrared spectrometer (FTIR), Model IRTracer 100. Figure 3 shows the functional groups present in PMHS, which were identified as low transmittance values. As expected, the vibration signal at 2966 cm^−1^ was due to the asymmetric stretching of the carbon-hydrogen (C-H) bonds of the CH_3_ group, the vibration signal at 2171 cm^−1^ corresponded to Si-H stretching, at 1408 cm^−1^ to the asymmetric bending of the Si-CH_3_ bond, at 1261 cm^−1^ to the Si-CH_3_ symmetric bending vibration, at 1126 cm^−1^ to the asymmetric Si-O-Si stretching, at 833 cm^−1^ to scissor bending of the Si-H bond, and at 763 cm^−1^ to the Si-C stretch vibration [44].

### 2.2. Experimental Methodology

An experimental design was determined to optimize the experiments, selecting independent variables and their corresponding levels. For this study, four factors were selected at three levels: (1) PMHS dosage (D); (2) stirring or homogenization time (H); (3) drying temperature after setting (T); and (4) percentage of replacement of CaSO_4_*0.5[H_2_O] by PET (RP). The experimental procedure followed a cube-centered experimental design. Table 3 shows the three levels of each experimental parameter. The PMHS dosage range was selected according to the recommendations of the manufacturer and the results obtained in a previous study [42]. The ranges of homogenization time (H) and drying temperature after setting (T) were selected to replicate industrial manufacturing conditions. A detailed explanation of the selection of H and T ranges can be found in a previous study [42]. The PET replacement was limited to a maximum of 10% because in the trial mixture, it was observed that larger PET replacements produced a negative effect on the fluidity, affecting the correct mixing of all the plasterboard components.

Table 4 shows the experimental design matrix. In this research, twelve experimental runs were evaluated. Considering the variability of the process, eight replicates under each condition were carried out to improve the reliability. Trials with PMHS without PET with factors D, H, and T at three levels were considered for the reference or baseline. The levels selected for factors D, H, and T in the cube-centered factorial experimental design were based on industry practice. Traditional plasterboard fabrication considers a homogenization process at speeds of 350–380 rpm, with mixing times between two and eight seconds, and oven drying at a gradually decreasing temperature starting at 315 °C and finishing at 178 °C [45].

A water/CaSO_4_·0.5[H_2_O] ratio of 0.95 was established for all tests because this ratio allows mixtures with PET to reach the proper incorporation of the components. Water and PMHS mixed manually were added in the mixer container. Then, CaSO_4_·0.5[H_2_O] mixed with PET was incorporated. An OSTER XPERT BLST3A-CPG052 Sunbeam Oster de Acuña, S.A. de C.V. Cd. Acuña, Coahuila México, mixer was used to reach the industrial range with a rotor blade radius of 2–3 cm at an angular velocity of 10,000 rpm. Stirring time was augmented 10–30 times to replicate industrial conditions [46]. To replicate the mixing conditions inside an industrial mixer, the linear velocity of the laboratory mixer’s blades was equalized to the angular velocity times the radius of the industrial mixer. Industrial drying was imitated with a laboratory oven to obtain forced convection drying. Forced convection ensured the removal of residual water without affecting the CaSO_4_·2[H_2_O] phases [47,48].

The mixtures were agitated for the time indicated in the experimental design matrix. Subsequently, they were poured into 30 × 26 × 8 mm silicone molds, demolded after 30 min, and placed to dry in the oven for 15 min at the drying temperature according to the design matrix (see Table 4). Samples were then kept in the oven at 40 °C for 19 h and kept in a conditioned room (21 ± 2 °C and 51 ± 7% RH) until used.

The effect of RP on the CaSO_4_·2[H_2_O] crystal morphology was characterized by determining the crystal length/width ratio and particle size distribution. The length/width ratio was determined using images captured with FESEM-EDS FEI QUANTA FEG 250 scanning electron microscopy (SEM) equipment, FEI, Hillsboro, Oregon, United States. Images were analyzed with openly available ImageJ software ImageJ vers.1.53k/Java 1.8.0_172 that included a feature to determine the distance between two selected points.

To assess the pore distribution, the hardened samples were subjected to X-ray microcomputed tomography (XMT), which generated 3D digital models that allowed us to visualize the interior of the samples [25,49,50,51]. For this study, a high-resolution SkyScan 1272 XMT device (Bruker, Kontich, Belgium) was used at 80 kV, 125 mA, with a rotation step of 0.4°, a 0.25 mm aluminum filter, and 12 µm voxel size. 3D images were obtained using NRecom reconstruction software (Bruker, Belgium). Images were reordered in space using DataViewer software (Bruker, Belgium) to standardize sample positioning. Thus, within a volume of interest (VOI) in the transverse plane of around 2 cm^3^, a quantitative evaluation was performed using CTan analysis software (Bruker, Belgium). Furthermore, images and videos of each sample were obtained using CTVox visualization software (Bruker, Belgium). Pixel size was 24 μm resolution. The threshold parameters used were lower grey 33 and upper grey 255.

The effect of RP on paste fluidity was measured according to standard UNE-EN 13279-2 Gypsum binders and gypsum plasters [52]. In addition, the effects on humidity resistance were measured by the mass percentage of water absorption, which was calculated using the following equation:

(1) $$A\;\left(\%\right)=\frac{W_{wet}-W_{dry}}{W_{dry}}\cdot 100\%$$

where *A* is the water absorption, $W_{wet}$ is the weight of the wet sample, and $W_{dry}$ is the weight of the dry sample.

Water absorption (A, in percentage) and flexural strength (Rf, in N) were measured following the standard UNE-EN 520 Gypsum plasterboards- [53]. Thermal conductivity (Ct, in W/(m·°K))) was measured with HotDisk TPS 1500 equipment that followed the standard UNE-EN ISO 22007-2 Plastics-Determination of thermal conductivity and thermal diffusivity [54]. In addition, the total porosity (Po), final crystalline structure of calcium sulfate dihydrate, and morphology, measured as the length-to-width ratio (L/W) of the CaSO_4_·2[H_2_O] crystals, were quantified. For additional information regarding the testing procedure, the reader is referred to our previously published work [42].

### 2.3. Experimental Design

To measure the performance of the PMHS plasterboard with and without PET, tests with both prototypes were performed based on the factorial experimental design shown in Table 3. Table 4 shows the details of each test and each experiment in the cube-centered factorial experimental design matrix. Each test was replicated eight times, and results were evaluated statistically to determine whether there was a significant difference between the mean performance values by applying a paired *t*-test. Overall, twelve experimental runs with eight replicates were performed (96 experiments in total). Assays N1, N2, and N3 did not involve PET content and were the baseline. Trials RP1–RP9 assessed the sensitivity of adding PET to the baseline. Statistical software Minitab^®^ version 19.1.1.0 (Minitab, LLC, State College, PA, USA) was used for the data analysis and processing.

## 3. Results and Discussion

### 3.1. Impact of PET on Mixture Fluidity

**Figure 4:**
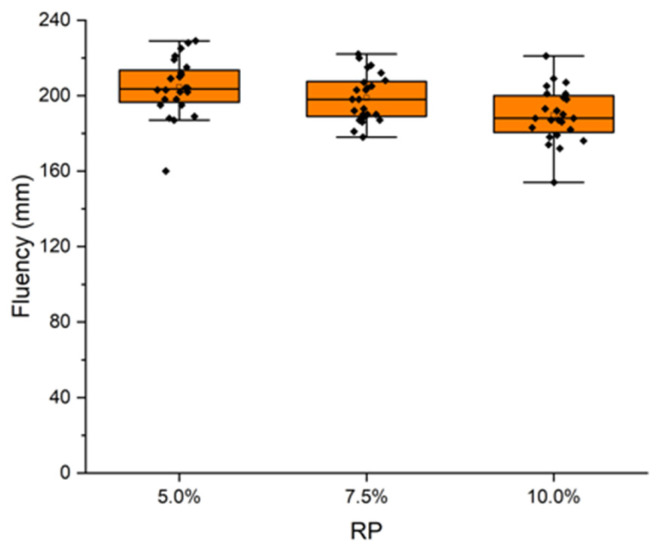
Mixture fluidity at different PET percentages.

Figure 4 shows the paste fluidity measurement using the standard UNE-EN 13279-2 Gypsum binders and gypsum plasters. Par 2: test methods [52]. The fluidity of the different mixtures with recycled PET microplastics decreased as the percentage of recycled PET microplastics in the base mixture increased. In fact, adding more than 10% PET in weight caused a loss of fluidity, making the mixing of the components difficult. Hence, a maximum replacement (RP) of 10% weight was defined.

### 3.2. Design Matrix Test Results

Table 5 and Table 6 show the results of all tests under the cube-centered factorial experimental design matrix. Table 5 shows the average measured in the eight replicates of each test and the standard deviation for the measured value of water absorption (A), flexural strength (Rf), and thermal conductivity (Ct). Adding recycled PET microplastics reduced the average water absorption from 3.5% to 2.3% for the tests with and without recycled PET microplastics for an overall reduction of 33%. The flexural strength results from tests without recycled PET microplastics presented an average of 123 N, and those with recycled PET microplastics 110 N, showing a decrease of 11%. Thermal conductivity tests without recycled PET microplastics presented an average of 0.308 W/m°K, while conductivity with recycled PET microplastics yielded, on average, 0.278 W/m°K, a 10% reduction.

Based on Table 5, a subgroup of runs was selected for crystal porosity and morphology analyses. The subgroup chosen for the mixes without PET at the minimum, medium, and maximum D, H, and T levels corresponded to trials N1, N2, and N3. Likewise, the RP3, RP6, and RP9 tests involved the addition of 10% PET with the same processing conditions than N1, N2, and N3, respectively. The porosity and morphology results measured with XMT and SEM are shown in Table 6. The crystals formed after the addition of PET were slightly more compact than those without the addition of PET. As the dose of PMHS, stirring time, and drying temperature increased, more compact crystals with a lower L/W ratio were formed. Average values were obtained through 50 measurements with ImageJ software ImageJ vers.1.53k/Java 1.8.0_172. Likewise, trials with added PET yielded a significantly higher total porosity than those without PET.

### 3.3. Crystal Morphology

**Figure 5:**
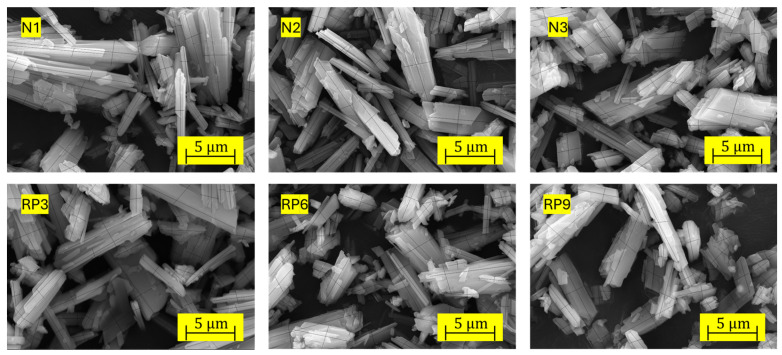
Crystal size variation (length/width) without and with recycled PET microplastics.

Figure 5 shows the morphologies of N1, N2, and N3 for the samples without recycled PET microplastics under conditions of the minimum, medium, and maximum levels of the factors, and RP3, RP6, and RP9 for samples with 10% PET under conditions of the minimum, medium, and maximum levels of factors, as defined in Table 3.

**Figure 6:**
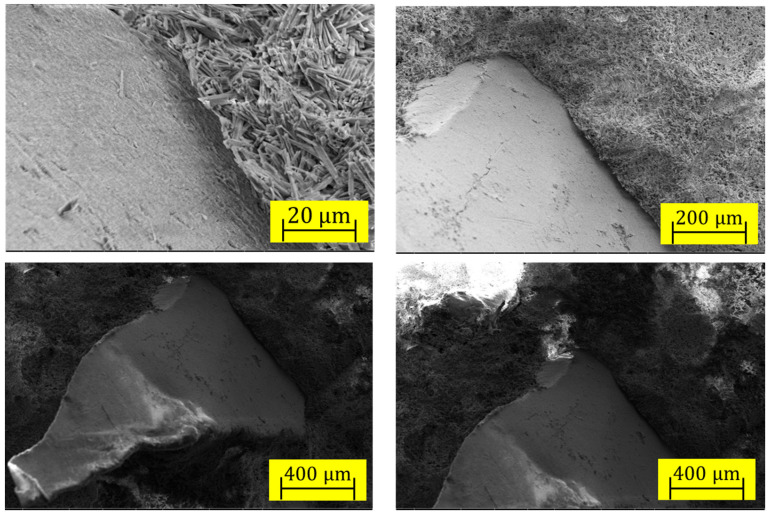
SEM images of ITZ in samples with recycled PET microplastics.

The morphology of the ITZ between the plastic residue and CaSO_4_*2[H_2_O] matrix can be seen in the SEM micrographs in Figure 6. The ITZ in some places showed a separation, suggesting fragility in those areas, while in others, the CaSO_4_*2[H_2_O] crystals were close to the surface of the recycled PET particle and the ITZ compact. The crystals formed a porous framework surrounding the much larger recycled PET particles. Continuous CaSO_4_*2[H_2_O]-PET junction surfaces with good adhesion and edges with little adhesion between the two phases were observed.

Finally, the statistical evaluation of the paired *t*-test of the samples without and with 10% RP at the medium level of dosage (D), stirring time (H), and drying temperature (T) (corresponding to the N2 and RP6 samples) yielded a *p*-value of 0.031, lower than 0.05, therefore, there was a significant difference in morphology when adding recycled PET microplastics. The test did not provide evidence for a significant impact on morphology at the minimum and maximum levels of D, H, and T (N1 and RP3; N3 and RP9).

### 3.4. Porosity

**Figure 7:**
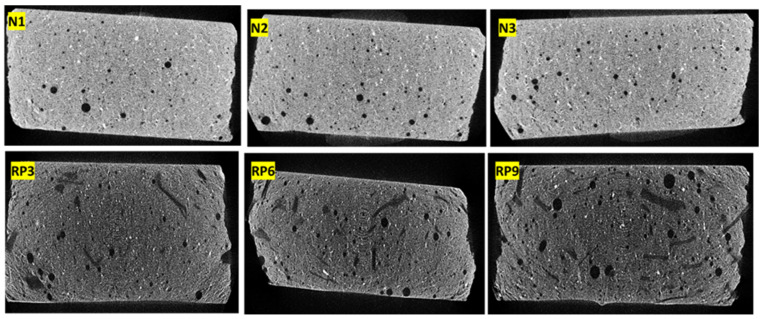
XMT 3D scan of plasterboard with PMHS without and with recycled PET microplastics.

Figure 7 shows the images of the 3D X-ray microtomography scan of samples with PMHS and without and with 10% RP manufactured under conditions of factors D, H, and T at the minimum, medium, and maximum levels as defined in Table 5. Black pores of several sizes and white points of calcium carbonate were observed. RP3, RP6, and RP9 also show the PET in a dark gray color, which was distributed randomly between the pores and the white particles. In addition, samples with 10% PET in weight showed larger air pores.

**Figure 8:**
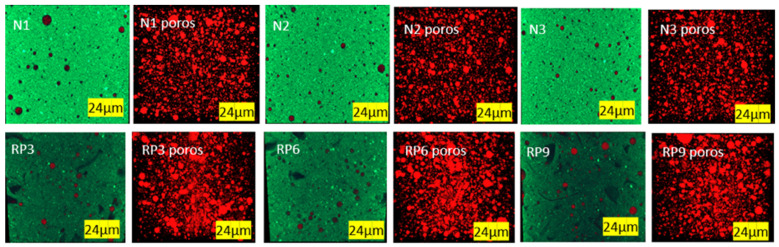
XMT 3D visualization of samples with PMHS without and with 10% of recycled PET microplastics.

Figure 8 shows the same samples where air pores were identified via image analysis in red. Image segmentation allowed us to evaluate the porosity quantitatively.

Table 6 shows the total porosity values of samples with PMHS, without and with 10% RP in the three combinations of levels of factors D, H, and T. The total porosity changed significantly with the addition of recycled PET microplastics, which generated voids within the mixtures, reaching maximum mean porous sizes at maximum values of D, H, and T (RP3, RP6, and RP9).

Results were analyzed with Minitab to determine the difference in trapped air pores when comparing samples without and with 10% recycled PET microplastics at the different levels of factors evaluated. The statistical analysis results (i.e., hypothesis test—paired *t*-test—Minitab) indicated a *p*-value of 0.001, less than 0.05, suggesting that the pore levels differed significantly due to the addition of PET.

**Figure 9:**
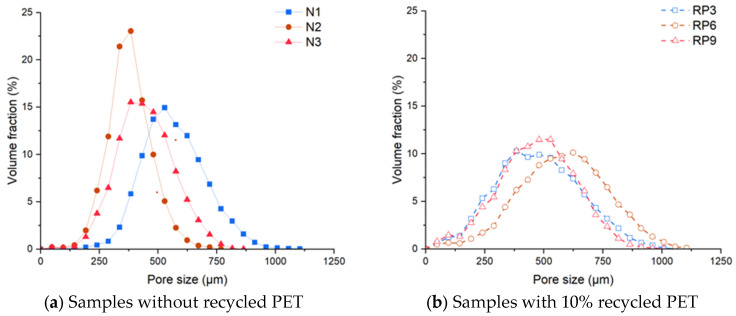
Pore size distribution of samples without recycled PET (**a**) and samples with recycled PET (**b**).

Figure 9 shows the pore size distributions based on the analyzed images. Figure 9a shows the results of pastes with PMHS and without recycled PET microplastics, while Figure 9b shows the results of pastes with PMHS and 10% RP in the three manufacturing conditions (i.e., D, H, and T at the minimum, medium, and maximum levels). Adding recycled PET microplastics to the plasterboards will increase the air pore size, mainly under the medium level conditions. The results suggest an optimal dose that maximizes the pore size in the existing CaSO_4_*2[H_2_O] matrix.

### 3.5. Water Absorption

**Figure 10:**
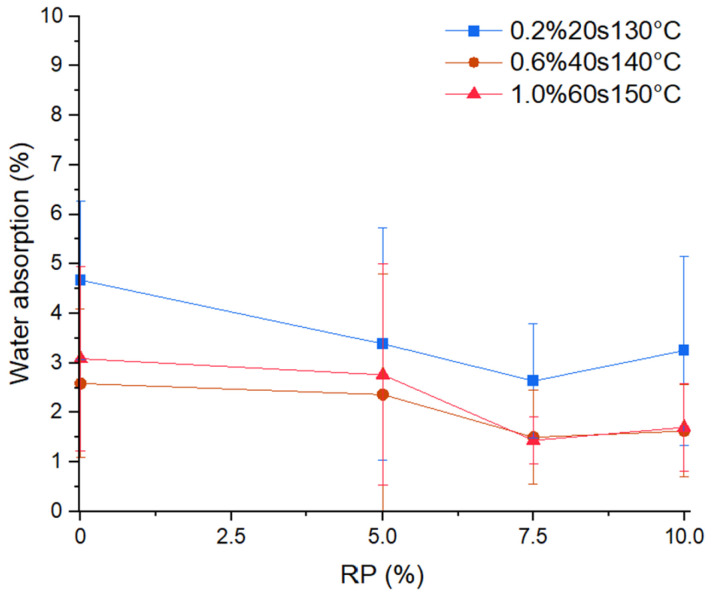
Effect of PET variation on water absorption.

Figure 10 shows the effect of replacing plasterboard material with PET on the water absorption. The water absorption slightly decreased with the addition of recycled PET microplastics. Statistical evaluation with the paired *t*-test for sets without and with an RP of 10% yielded a *p*-value of 0.014, indicating that the addition of 10% of recycled PET microplastics allows for a reduction in the water absorption of plasterboards with PMHS.

This decrease in the water absorption capacity of plasterboard with PET content resulted from the effect on the crystal morphology and porosity caused by the variety of factors D, H, and T, as evidenced in Section 3.3 and Section 3.4. The structure of the CaSO_4_*2[H_2_O] that formed was more compact and increased the empty spaces in the matrix. The recycled PET microplastics worked as a barrier, making it difficult for water molecules to enter the mixture. Another reason for inhibiting water absorption relates to the lower CaSO_4_*2[H_2_O] mass. The samples reacted well against water because of the PET waterproofing conditions [34] and the hydrophobic effect of the PMHS admixture.

Statistical evaluation was carried out with the *t*-test for two mean samples, the three sets without PET, and the nine sets with PET addition, as shown in Table 5, yielding a *p*-value of 0.033, lower than 0.05. Therefore, there was a significant difference in the mean water adsorption value between both groups. Furthermore, the water absorption of plasterboard with 0.2% doses of PMHS presented a larger water absorption than plasterboards with 0.6% and 1.0%. This latter result was similar for the samples without and with recycled PET.

### 3.6. Flexural Strength

**Figure 11:**
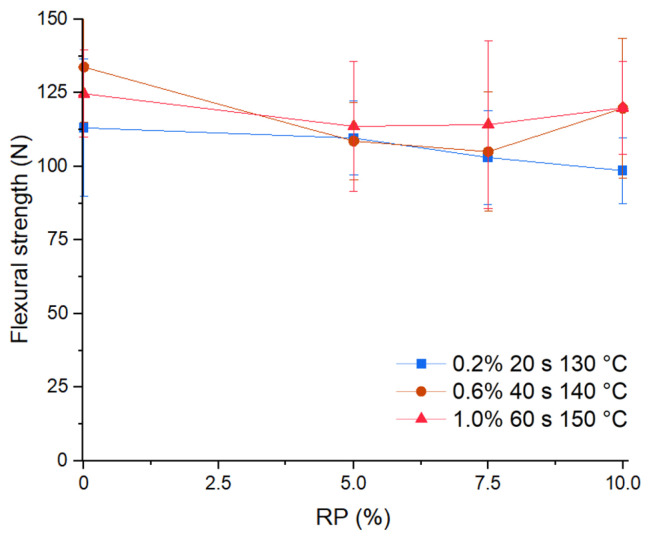
Effect of RP on flexural strength.

The addition of PET slightly decreased the flexural strength (Rf). Figure 11 presents the effect of RP on the flexural strength of the pastes. Rf decreased for an RP of 5%, the drop being greater at the minimum and medium levels of the experimental factors (dosage, stirring time, drying temperature). Between an RP of 7.5% and 10%, the Rf did not present significant differences between the flexural strength in the three scenarios.

Statistical evaluation was performed with the *t*-test for two samples, for the three sets without PET, and the nine sets with PET, as shown in Table 5, yielding a *p*-value of 0.015, suggesting that the flexural strength of plasterboard with the addition of PET presents a 11% lower bending capacity than a plasterboard without PET.

Statistical evaluation was carried out with the paired *t*-test for the sets without and the subgroup of those samples with 10% PET addition, as shown in Table 5. The test results showed a *p*-value of 0.072, above 0.05, suggesting that the resistance to bending of plasterboards without and with a 10% PET addition did not present a significant difference.

The decrease in flexural strength might be explained by the ITZ analysis on the images, which highlight areas with good adherence between the recycled PET microplastics and the CaSO_4_*2[H_2_O] matrix and other low adherence factors, confirming the results of a previous study that incorporated other plastics into the matrix [34]. In addition, the larger air pore size resulting from adding PET produced brittleness and discontinuities in the joints. This was also observed in another study that compared the flexural strength between samples with the addition of other plastics and without addition, thus only with CaSO_4_*0.5[H_2_O] [35]. Although the Rf decrease was weaker at some levels of D, H, and T, it still fulfilled the bending strength requirement of standard ASTM C1396/C1396M, 2017 Specification for Gypsum Board, so we conject that the PMHS that reacts with water during the hydration from CaSO_4_*0.5[H_2_O] to CaSO_4_*2[H_2_O] may also form a film on the surface of PET located around the particles. This development would increase the adhesion between the CaSO_4_*2[H_2_O] matrix and PET pieces, contributing to the effects on bending resistance and agreeing with a study using a different additive [38].

### 3.7. Thermal Conductivity (W/m°K)

**Figure 12:**
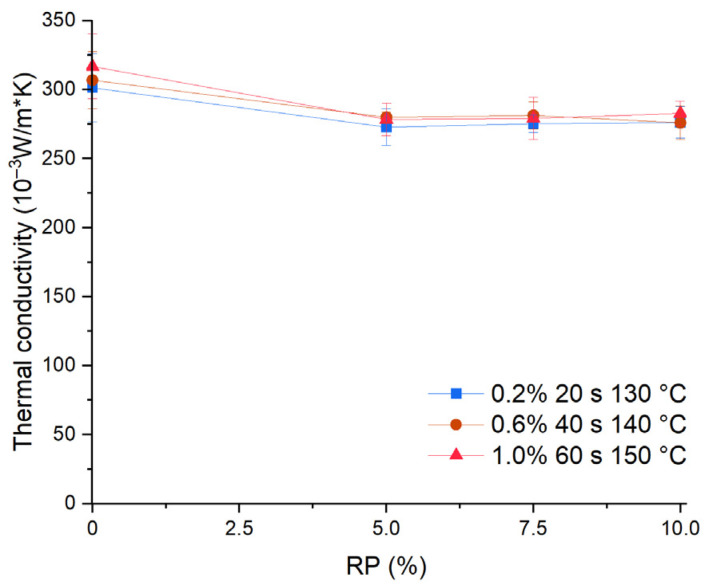
Effect of RP on thermal conductivity.

The addition of recycled PET microplastics decreased the thermal conductivity. Figure 12 shows the effect of RP on the thermal conductivity of the pastes. Under the three conditions, Ct decreased when recycled PET microplastics was added up to 5%. However, 10% additions of recycled PET microplastics did not produce a significant effect on thermal conductivity.

Statistical evaluation was carried out with the *t*-test for two mean samples, for the three sets without PET, and the nine sets with PET, using the definitions shown in Table 5. The *p*-value was 0.000, less than 0.05, indicating that the thermal conductivity of plasterboard with PMHS presented a significant difference between samples without and with PET. The thermal conductivity average of samples with 5% or more recycled PET microplastic was 10% lower compared to the average of samples without recycled PET microplastics.

Statistical evaluation with the paired *t*-test for the sets without and with 10% of recycled PET microplastics led to a *p*-value of 0.008, indicating that the thermal conductivity of plasterboards with PMHS significantly differed according to PET content, and the samples with PET showed 10% lower thermal conductivity than samples without 10% PET.

The results show that PET improved the thermal performance, corroborating studies that focused on other polymer residues [55]. The elaborate microstructural pattern of recycled PET microplastics resulted in air voids facilitated by higher surface tension between the plastic and water [56]. This behavior may also result from thermal conductivity, having been affected by the pore volume of the samples and recycled PET microplastics, which reduces the thermal conductivity [55]. The conduction heat transfer pathway through the plasterboards takes a lot of work [57]. By incorporating microplastics, the mass of CaSO_4_*2[H_2_O], water, and PMHS was lower, so the thermal conductivity values will be lower because the thermal conductivity of PET is 0.24 W/m°K [58]. The mixture without PET varied between 0.301 and 0.317 W/m°K, while the mixture with PET varied between 0.273 and 0.283 W/m°K.

## 4. Conclusions

This paper analyzed the hygrothermal and mechanical performance of plasterboards with added polymethylhydrosiloxane (PMHS) and recycled polyethylene terephthalate (PET) microplastic residues. The variation of four preparation factors (PMHS dosage, homogenization or stirring time, drying temperature after setting, and replacement of CaSO_4_*0.5[H_2_O] by recycled PET microplastics) was evaluated in terms of the plasterboard sample performance obtained in water adsorption, flexural strength, and thermal conductivity. Changes in crystal morphology and the porosity of the former plasterboard were observed, therefore altering the water absorption capacity, flexural strength, and thermal conductivity. These results show that the addition of recycled PET in plasterboard mixtures is a promising alternative. The main conclusions are listed below:

- The replacement of CaSO_4_*2[H_2_O] with recycled PET microplastics reduced the plasterboard fluidity. In fact, replacements beyond 10% in weight will make it considerably difficult to obtain the correct mixing of raw materials.
- There were significant differences in the morphology of CaSO_4_*2[H_2_O] between samples with and without recycled PET microplastics, which influenced the quality of the interfacial transition zone of the plasterboards.
- Replacement of up to 10% of CaSO_4_*0.5[H_2_O] with recycled PET microplastics did not produce a significant reduction in the water absorption and flexural strength of the plasterboard.
- Replacement of CaSO_4_*0.5[H_2_O] with 5% recycled PET microplastics in weight reduced the thermal conductivity of plasterboards by around 10%. However, larger replacements did not produce significant additional reductions in the thermal conductivity.
- The primary use of plasterboard is to build partitions, wall linings, or ceilings in building. Introducing recycled PET microplastics into its composition presents a promising alternative for enhancing its performance. By integrating recycled PET, not only does the plasterboard maintain its flexural strength and water resistance, but it also sees an improvement in thermal insulation capabilities.

## Figures and Tables

**Table 1:** Chemical composition of the calcium sulfate hemihydrate (%).

SO_3_	CaO	SiO_2_	Al_2_O_3_	Fe_2_O_3_	SrO	K	LOI
45.00	39.83	1.10	0.50	0.18	0.17	0.05	13.18

**Table 2:** Mineralogical composition of the calcium sulfate hemihydrate.

Mineral	Formula	Fraction (%wt)
Bassanite	CaSO_4_·0.5H_2_O	83.2
Calcite	CaCO_3_	10.8
Anhydrite	CaSO_4._	6.0

**Table 3:** The experimental factors and levels.

Experimental Factors	Levels		
	-	0	+
PMHS doses, D (%weight)	0.2%	0.6%	1.0%
Homogenization time, H (s)	20	40	60
Drying temperature, T (°C)	130	140	150
PET replacement, RP (%weight)	5.0%	7.5%	10.0%

**Table 4:** Experimental design matrix. Mixes of calcium sulfate hemihydrate with water, PMHS, and without/with PET.

	Run	D (%)	H (s)	T (°C)	RP (%)	Water/CaSO_4_*0.5[H_2_O]	Water Absorption (%)	Bending Strength (N)	Thermal Conductivity (W/m°K)	SEM	XMT
Without PET	N1	0.2	20	130	0.0	0.95	X	X	X	X	X
	N2	0.6	40	140	0.0	0.95	X	X	X	X	X
	N3	1.0	60	150	0.0	0.95	X	X	X	X	X
With PET	RP1	0.2	20	130	5.0	0.95	X	X	X		
	RP2	0.2	20	130	7.5	0.95	X	X	X		
	RP3	0.2	20	130	10.0	0.95	X	X	X	X	X
	RP4	0.6	40	140	5.0	0.95	X	X	X		
	RP5	0.6	40	140	7.5	0.95	X	X	X		
	RP6	0.6	40	140	10.0	0.95	X	X	X	X	X
	RP7	1.0	60	150	5.0	0.95	X	X	X		
	RP8	1.0	60	150	7.5	0.95	X	X	X		
	RP9	1.0	60	150	10.0	0.95	X	X	X	X	X

**Table 5:** Results of A, Rf, Ct, according to the experimental design matrix.

	Trial	D (%)	H (s)	T (°C)	RP (%)	A (%)		Rf (N)		Ct (W/m°K)	
						Avg	Dev	Avg	Dev	Avg	Dev
Without PET	N1	0.2	20	130	0.0	4.7	2.1	113	23	0.301	0.025
	N2	0.6	40	140	0.0	2.6	1.5	134	19	0.307	0.021
	N3	1.0	60	150	0.0	3.1	1.9	125	15	0.317	0.023
With PET	RP1	0.2	20	130	5.0	3.4	2.3	110	13	0.273	0.013
	RP2	0.2	20	130	7.5	2.6	1.2	103	16	0.275	0.006
	RP3	0.2	20	130	10.0	3.3	1.9	99	11	0.276	0.011
	RP4	0.6	40	140	5.0	2.4	2.4	109	13	0.280	0.004
	RP5	0.6	40	140	7.5	1.5	0.9	105	20	0.281	0.010
	RP6	0.6	40	140	10.0	1.6	0.9	120	24	0.276	0.012
	RP7	1.0	60	150	5.0	2.8	2.2	114	22	0.278	0.012
	RP8	1.0	60	150	7.5	1.4	0.5	114	29	0.279	0.015
	RP9	1.0	60	150	10.0	1.7	0.9	120	16	0.283	0.009

**Table 6:** Results of the L/W and Po tests in line with the experimental design matrix.

L/W and Po at minimum, medium, and maximum levels	**Trial**	**D (%)**	**H (s)**	**T (°C)**	**RP (%)**	**L/W**		**Po (%vol)**
						**Avg.**	**Dev.**	
	N1	0.0	20	130	0.0	3.64	2.57	1.090
	N2	0.0	40	140	0.0	3.71	1.77	1.458
	N3	0.0	60	150	0.0	2.71	1.34	1.818
	RP3	0.2	20	130	10.0	3.28	1.90	2.812
	RP6	0.6	40	140	10.0	2.92	1.86	3.023
	RP9	1.0	60	150	10.0	2.59	1.41	3.524

## Data Availability

Data will be available upon written request.
